# Octreotide-induced thrombocytopenia: a case report

**DOI:** 10.1186/1752-1947-5-286

**Published:** 2011-07-05

**Authors:** Armin Rashidi, Nahid Rizvi

**Affiliations:** 1 Department of Internal Medicine, Eastern Virginia Medical School, 825 Fairfax Avenue, Suite 410, Norfolk, VA 23507, USA; 2 Department of Internal Medicine, Hampton Veterans Affairs Medical Center, Hampton, VA 23667, USA

## Abstract

**Introduction:**

Thrombocytopenia is an extremely rare complication of octreotide therapy and can be life threatening in the setting of esophageal variceal bleeding. We report a case of octreotide-induced reversible thrombocytopenia in a 54-year-old Caucasian man with alcohol-induced cirrhosis and upper gastrointestinal bleeding.

**Case presentation:**

Our patient's platelet count dropped from 155,000/mm^3 ^upon admission to 77,000/mm^3 ^a few hours after initiation of octreotide therapy and stayed low until the drug's administration was discontinued. Significant recovery was achieved quickly after discontinuation of octreotide.

**Conclusions:**

Thrombocytopenia is a rare but potentially serious side effect of octreotide therapy and may complicate esophageal variceal bleeding. Physicians should be vigilant in identifying this potentially serious condition.

## Introduction

Drug-induced thrombocytopenia can complicate esophageal variceal bleeding. Octreotide is a standard treatment in patients with portal hypertension presenting with upper gastrointestinal bleeding. Octreotide-induced thrombocytopenia is a rare condition that has been reported in only two previous cases [1,2]. Another case is reported herein.

## Case presentation

A 54-year-old Caucasian man with a medical history of alcoholic liver disease and grade I esophageal varices presented to our hospital with a one-day history of hematemesis and light-headedness. The patient did not have any comorbidities, and his last alcoholic beverage consumption was three days before admission. His initial vital signs revealed blood pressure of 111/73 mmHg, heart rate of 129 beats/minute, respiratory rate of 22 breaths/minute, and 100% oxygen saturation on room air. His physical examination revealed mild scleral icterus, gynecomastia, ascites, hepatomegaly, and palmar erythema. His relevant laboratory findings were hemoglobin 11.1 g/dL, platelets 155,000/mm^3^, International Normalized Ratio 1.4, and mean corpuscular volume 89.9 fL/red blood cell.

**Figure 1:**
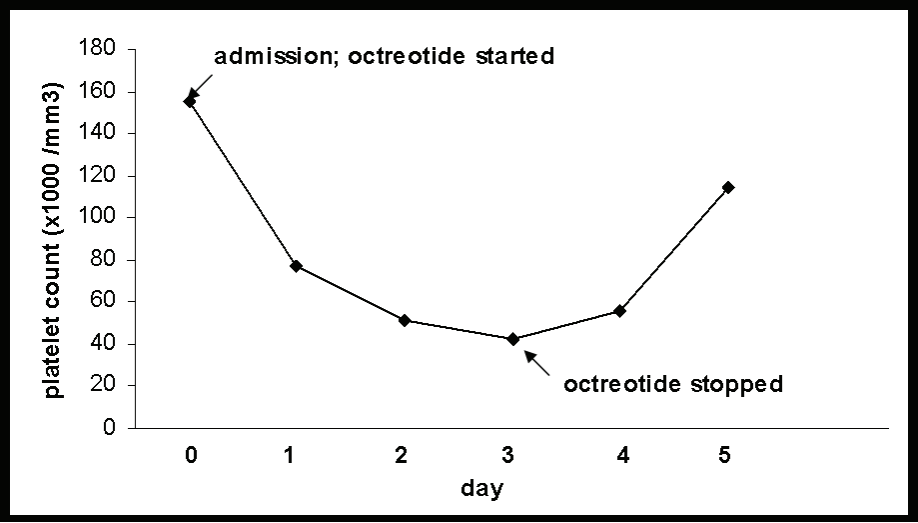
**Platelet count during the course of hospitalization**. The patient's platelet count dropped by about 50% immediately following octreotide administration upon admission and recovered after octreotide was discontinued three days later.

The patient received 2 L of normal saline, 2 U of packed red blood cells, a 50 μg octreotide bolus intravenous injection followed by continuous infusion at 50 μg/hour, pantoprazole 80 mg bolus infusion, and thiamine and folic acid administered intravenously, along with ciprofloxacin. His bleeding stopped and esophagogastroduodenoscopy revealed non-bleeding grade I esophageal varices. Nine hours after admission the patient's platelet count had decreased to 77,000/mm^3 ^and stayed around 50,000/mm^3 ^for 3 days following admission (Figure 1). Evaluations for acute thrombocytopenia, including a peripheral blood smear and a disseminated intravascular coagulation panel, did not show any abnormalities. Octreotide was discontinued 72 hours after admission, with a presumptive diagnosis of drug-induced thrombocytopenia. A quick recovery in the patient's platelet count occurred, and he remained stable and was discharged on day five after admission with a platelet count of 114,000/mm^3^. While other medications such as antibiotics and proton pump inhibitors were administered during his hospitalization, his platelet count decreased after octreotide initiation and increased only after octreotide was discontinued. A diagnosis of octreotide-induced reversible thrombocytopenia was made.

## Discussion

Thrombocytopenia is an extremely rare side effect of octreotide therapy. To our knowledge, only two cases of this condition have previously been reported in the literature.

In the first case, the platelet count in a 53-year-old man with alcohol-induced cirrhosis dropped immediately after octreotide administration from 144,000/mm^3 ^to 75,000/mm^3 ^and continued to decrease within the next 50 hours to 4000 despite multiple platelet transfusions. After octreotide was discontinued, the patient's platelet count gradually recovered to 28,000/mm^3 ^within about two days. Inadvertent octreotide administration on a subsequent admission resulted in an immediate drop in platelets from 214,000/mm^3 ^to 89,000/mm^3 ^[1]. In the second reported case, that of a 42-year-old woman with hepatitis C- and alcohol-induced cirrhosis, the patient's platelet count dropped immediately from 122,000/mm^3 ^to 72,000/mm^3 ^following octreotide administration [2]. In both of these two cases as well as in our patient, octreotide was administered as a standard 50 μg bolus. Interestingly, in all three cases, the immediate drop in platelets was about 50%.

The mechanism of drug-induced thrombocytopenia is most often immunologic [3], that is, accelerated platelet destruction by drug-dependent antibodies binding to platelet surface glycoproteins [4]. The median recovery time from drug-induced thrombocytopenia following drug discontinuation is thought to be about 1 week [5].

## Conclusions

Physicians need to be aware of the possibility of octreotide-induced thrombocytopenia. Although rare, this condition may significantly worsen esophageal variceal bleeding in patients with cirrhosis. Continued bleeding not explained by anemia and/or clotting factor deficiencies alone should immediately prompt clinical suspicion of octreotide-induced thrombocytopenia. We recommend serial monitoring of not only hemoglobin but also platelets in patients with esophageal variceal bleeding treated with octreotide. On the basis of the limited data available, the level of suspicion for octreotide-induced thrombocytopenia should be high, especially if the immediate drop in platelets is about 50%.

## Consent

Written informed consent was obtained from the patient for publication of this case report and any accompanying images. A copy of the written consent is available for review by the Editor-in-Chief of this journal.

## Competing interests

The author declares that they have no competing interests.

## Authors' contributions

AR and NR collected and interpreted the data. AR wrote the first draft of the manuscript, and NR revised it. Both authors approved the final draft.
